# Protein Z and Protein Z Complex in the Acute Phase of Ischemic Stroke: Potential Markers of Coagulation and Prognostic Value in Patients Treated with Thrombolysis or Conservative Therapy

**DOI:** 10.3390/neurolint18020029

**Published:** 2026-02-06

**Authors:** Małgorzata Wiszniewska, Urszula Włodarczyk, Mariusz Domagalski, Artur Słomka, Inga Dziembowska, Maciej Gawrysiak, Anna Żdanowicz, Ewa Żekanowska

**Affiliations:** 1 Emergency Medical Services, University of Applied Sciences, 64-920 Piła, Poland; mpwisz@gmail.com; 2 Neurological Department with Stroke Unit, Specialist Hospital, 64-920 Piła, Polandmariuszdom.75@hotmail.com (M.D.); 3 Department of Pathophysiology, Ludwik Rydygier Collegium Medicum, Nicolaus Copernicus University in Toruń, 85-094 Bydgoszcz, Poland; artur.slomka@cm.umk.pl (A.S.); i.dziembowska@gmail.com (I.D.); zorba@cm.umk.pl (E.Ż.); 4 Department of Nursing, Stanislaw Staszic State University of Applied Science, 64-920 Piła, Poland; azdanowicz@ans.pila.pl

**Keywords:** protein Z, protein Z complex, acute ischemic stroke, thrombolysis

## Abstract

Background/Objectives: Protein Z (PZ) and the protein Z-dependent protease inhibitor (ZPI) are vitamin K-dependent regulators of coagulation that inhibit activated factor Xa. Their relevance in ischemic stroke (IS) remains insufficiently characterized, with inconsistent evidence regarding their association with stroke severity and outcomes. This study aimed to evaluate the concentrations and dynamics of PZ and ZPI in the acute phase of IS in patients treated with intravenous thrombolysis or conservative therapy and to assess their potential prognostic significance. Methods: Eighty-four patients with acute IS were enrolled and divided into two groups: group I treated with intravenous thrombolysis (rt-PA) and group II managed conservatively. PZ and ZPI concentrations were measured using ELISA on admission (day 1) and on day 7. Associations with factor X activity, the modified Rankin Scale (mRS), and the National Institutes of Health Stroke Scale (NIHSS) were analyzed using nonparametric tests and Spearman correlations (*p* < 0.05). Results: PZ concentrations were significantly higher in thrombolysis-treated patients than in conservatively managed patients both on day 1 (median: 2810.05 vs. 2178.50 ng/mL; *p* = 0.024) and day 7 (2982.90 vs. 2286.50 ng/mL; *p* = 0.026). A slight negative correlation between PZ and mRS on day 7 was observed in the conservative group (r = −0.360; *p* = 0.043). In thrombolysis-treated patients with dyslipidemia, PZ increased from day 1 to day 7, whereas it decreased in those without dyslipidemia. No significant correlations were found between PZ, ZPI, or factor X and NIHSS or ASTRAL scores. Conclusions: Higher PZ concentrations in the acute phase of IS—particularly in rt-PA-treated patients—may reflect differences related to the timing of the acute ischemic process and reperfusion status, suggesting potential utility as markers of stroke severity or outcome.

## 1. Introduction

Ischemic stroke (IS) occurs as a result of vessel occlusion by a thrombus [1,2]. Intravenous thrombolytic treatment with recombinant tissue plasminogen activator (t-PA) continues to be the gold standard for the treatment of the acute phase of IS [3]. New biomarkers of ischemic stroke (IS) are continuously being sought to improve the understanding of its pathogenesis, as well as to enhance diagnosis and prognosis. [4,5]. The essence of blood coagulation is the conversion of soluble plasma fibrinogen into a three-dimensional fibrin network that stabilizes the platelet hemostatic plug [6]. This process involves plasma proteins, tissue factor (TF) contained in cell membranes, membrane phospholipids, and calcium ions [4]. One important but relatively less recognized component of hemostasis is protein Z, a key element of the protein Z system [7]. Protein Z is a vitamin K-dependent plasma glycoprotein that, together with the protein Z-dependent protease inhibitor (ZPI), plays an important regulatory role in coagulation by inhibiting activated factor Xa and modulating thrombin generation [8,9]. The protein Z system, also referred to as the protein Z complex, consists of protein Z, the protein Z-dependent protease inhibitor (ZPI), and factor Xa. The protein Z complex promotes the inactivation of activated factor Xa, thereby inhibiting coagulation through this pathway. ZPI also has the ability to degrade factor XI [7,8,9]. However, there is no consensus regarding the influence of protein Z concentrations on coagulation processes in patients with acute IS or during the recovery phase [10,11]. Some investigators report an association between protein Z levels and the occurrence of IS, whereas others do not confirm such a correlation [11,12,13,14].

To date, data directly comparing short-term changes in protein Z and protein Z-dependent protease inhibitor levels between thrombolysis-treated and conservatively managed acute ischemic stroke patients remain limited. The objectives of the study were as follows: to analyze the levels and dynamics of protein Z concentrations in the acute phase of IS at admission and on day 7 of hospitalization in patients treated with intravenous thrombolysis (study group 1) and those managed conservatively (study group 2); to determine the correlation between protein Z concentrations and clinical outcomes assessed using the modified Rankin Scale (mRS) and the National Institutes of Health Stroke Scale (NIHSS) in patients with acute IS; to evaluate the correlations between protein Z concentrations and selected coagulation parameters in the acute phase of IS; and to assess the clinical utility of measuring protein Z concentrations in patients treated with thrombolysis and conservative therapy.

## 2. Materials and Methods

The study group consisted of 84 patients with acute IS treated in the Neurology Department with Stroke Unit in the Stanislaw Staszic Specialist Hospital in Piła in the period from 2016 to 2019.

IS was diagnosed based on a CT scan performed upon the patients’ arrival at the hospital (the longest waiting time for the scan was 20 min). The scan results were immediately assessed by a radiologist.

Patients were divided into two groups based on eligibility for intravenous thrombolysis. The inclusion criterion for thrombolytic treatment was based on the guidelines applicable at the time [2].

Study group I consisted of patients treated with intravenous recombinant tissue plasminogen activator (rt-PA, alteplase) according to current clinical guidelines, within 4.5 h from stroke symptom onset. Antiplatelet therapy with acetylsalicylic acid (ASA) at a dose of 150 mg daily was initiated 24 h after completion of thrombolytic therapy, following control neuroimaging.

Study group II comprised patients managed conservatively, who were not eligible for intravenous thrombolysis, mainly due to presentation beyond the therapeutic time window (>4.5 h from symptom onset). Conservative treatment in the acute phase included antiplatelet therapy with ASA at a dose of 150 mg daily and intravenous fluid therapy adjusted to individual cardiovascular status, typically ranging from 1000 to 2000 mL per day.

All patients were evaluated in a certified Neurology Department with a Stroke Unit. Clinical assessment included a structured medical history and physical examination performed at hospital admission and repeated on day 7 of hospitalization. Venous blood samples for laboratory analyses were obtained shortly after admission and again on day 7. Demographic characteristics and vascular risk factors, including age, body weight, waist–hip ratio (WHR), and blood pressure, were recorded for each patient. Clinical stroke subtype was classified according to the Oxfordshire Community Stroke Project (OCSP) criteria (TACI, PACI, LACI, POCI), and stroke etiology was determined using the TOAST classification [15,16]. Diagnostic workup included noninvasive vascular imaging with Doppler ultrasonography of the extracranial arteries, as well as standard cardiac assessment comprising electrocardiography, Holter monitoring, and transthoracic echocardiography.

Prior to initiation of intravenous thrombolysis, routine laboratory investigations were performed, including coagulation parameters, lipid profile, and calculation of the neutrophil-to-lymphocyte ratio. Routine coagulation testing included assessment of factor X activity, while lipid profile analysis comprised total cholesterol, low-density lipoprotein cholesterol (LDL-C), high-density lipoprotein cholesterol (HDL-C), and triglycerides.

Protein Z and ZPI concentrations were determined using an enzyme-linked immunosorbent assay (ELISA). Protein Z was measured with the ZYMUTEST PZ kit (HYPHEN BioMed, SAS, Neuville-sur-Oise, France), with a detection threshold of ≤0.25 µg/mL. ZPI was measured using a kit from Cloud-Clone Corp (Houston, TX, USA), with a minimum detectable concentration of <0.00046 µg/mL. After centrifugation, plasma samples were separated and stored at −80 °C for a maximum period of six months prior to ELISA analysis, with repeated freeze–thaw cycles avoided.

Statistical analysis was performed using STATISTICA software, version 13.3 (TIBCO, Kraków, Poland). The normality of variable distributions was assessed with the Shapiro–Wilk test. Quantitative variables were presented as median (Me), minimum (Min.), and maximum (Max.) values, while categorical variables were presented as counts (N) and percentages.

For comparisons between groups, nonparametric tests were applied: the Mann–Whitney U test for two-group comparisons and the Kruskal–Wallis rank ANOVA test (with Dunn’s post hoc test) for quantitative variables. The chi-square test was used for categorical variables. Spearman’s correlation analysis was applied to evaluate monotonic relationships between variables. A *p*-value of <0.05 was considered statistically significant.

### Limitations

An important limitation of this study is the difference in time from stroke onset between the thrombolysis-treated and conservatively managed groups at the time of the first blood sampling. As eligibility for intravenous thrombolysis is strictly time-dependent, patients assigned to conservative treatment were, by definition, examined at a later stage of the ischemic process. Therefore, the observed differences in protein Z and ZPI concentrations may reflect not only treatment-related effects but also differences in the temporal dynamics of endogenous coagulation and fibrinolytic processes during the acute phase of ischemic stroke. Accordingly, comparisons between the groups should be interpreted in the context of these physiological time-dependent processes.

The relatively small sample size and short observation period (limited to seven days) may not fully capture longer-term changes in protein Z and ZPI concentrations. Moreover, the study did not include a healthy control group, as all participants had acute ischemic stroke; thus, comparisons were restricted to two stroke cohorts differing in treatment pathway and time from symptom onset, precluding direct assessment of baseline protein Z levels. Finally, imaging-based parameters such as thrombus burden, recanalization status, or hemorrhagic transformation were not analyzed and should be addressed in future studies.

## 3. Results

The study population comprised 84 patients with acute ischemic stroke, including 52 individuals treated with intravenous thrombolysis (group I) and 32 patients managed without thrombolysis (group II). The proportion of women was comparable between the two groups, accounting for 42.3% of patients in the thrombolysis group and 41.5% in the conservatively treated group, with no statistically significant difference observed.

Baseline demographic parameters, including age, body weight, height, and waist–hip ratio (WHR), were similar in both groups, and no significant intergroup differences were identified (Table 1).

Stroke subtype distribution according to the Oxfordshire Community Stroke Project (OCSP) classification showed that lacunar infarction was the most frequent clinical presentation in both groups, occurring in 38.5% of patients treated with thrombolysis and 37.5% of patients treated conservatively. Total anterior circulation infarction (TACI) was the least common subtype in both cohorts.

Analysis of stroke etiology based on the TOAST classification revealed a higher proportion of cardioembolic strokes in the conservatively treated group (37.5%) compared with the thrombolysis group (23.1%), although this difference did not reach statistical significance. Other etiological categories, including large-artery atherosclerosis, small-vessel disease, and strokes of undetermined etiology, were distributed comparably between the two groups.

A detailed summary of demographic characteristics, clinical stroke subtypes, and etiological classifications for both study groups is presented in Table 1.

The concentration of protein Z on the first day after stroke onset was significantly higher in group I compared with group II (Table 2).

Protein Z-dependent protease inhibitor (ZPI) levels on day 1 were significantly lower in study group I than in study group II (Table 3).

Factor X concentration on the first day after stroke onset was similar in both groups (Table 4).

On the seventh day after stroke onset, the concentration of protein Z in study group I was significantly higher than in study group II (Table 5).

On the seventh day after stroke onset, the concentration of the protein Z-dependent protease inhibitor (ZPI) in group I was significantly lower than in group II (Table 6).

On the seventh day after stroke onset, factor X concentrations were similar in both study groups I and II (Table 7).

Table 8 presents the values of PZ, ZPI, and FX in study groups I and II on the first and seventh days. It shows that factor X concentration in group I decreased on day 7, whereas in group II, it increased; both differences proved to be statistically significant (*p* < 0.001). ZPI concentration in group I also decreased on day 7, but this difference was not statistically significant (*p* = 0.203). In group II, ZPI levels increased slightly, although not significantly (*p* = 0.531). As for protein Z, concentrations increased slightly in both groups on day 7, but the changes did not reach statistical significance (*p* = 0.465 for group I and *p* = 0.329 for group II).

On the day of hospital admission (day 1 of stroke), a higher PZ level was associated with a lower ZPI level, as indicated by a moderate negative correlation (r = −0.38, *p* < 0.001), and with a higher FX level, as indicated by a moderate positive correlation (r = 0.26, *p* = 0.021). Conversely, a higher ZPI level on day 1 was associated with a lower FX level, as indicated by a moderate negative correlation (r = −0.29, *p* = 0.01).

The correlations between PZ, ZPI, and FX at the second measurement (day 7) are presented in Table 9. A higher PZ level was associated with a higher FX level, and this relationship was statistically significant (*p* = 0.016).

The relationships between stroke etiology, defined according to the TOAST classification, and PZ concentrations in study groups I and II are presented in Table 10. Significantly lower PZ levels on day 7 were observed only in patients with extracranial large-artery atherosclerosis in group I (Me = 2110.40) compared with patients of undetermined etiology (Me = 3049.65, *p* < 0.004) and those with small-vessel disease (Me = 3749.80, *p* < 0.02). In group II, no statistically significant differences were found.

No significant differences in ZPI results were observed according to stroke etiology defined by the TOAST classification in the studied groups (Table 11).

No significant differences in FX results were observed according to stroke etiology defined by the TOAST classification, as presented in Table 12.

No significant differences in PZ and ZPI results were found between patients with cardioembolic and non-cardioembolic stroke.

No statistically significant differences in protein Z (PZ), protein Z-dependent protease inhibitor (ZPI), or factor X (FX) concentrations were observed in either study group with respect to smoking status, diabetes mellitus, or arterial hypertension.

Statistically significant differences were identified in subgroup analyses of patients with dyslipidemia and atrial fibrillation. In patients with dyslipidemia, protein Z concentrations on day 7 were significantly higher compared with day 1 and were also higher than those observed in patients without dyslipidemia (group I, *p* = 0.03; Table 13). Similarly, in patients with dyslipidemia, ZPI concentrations on day 7 were significantly lower compared with patients without dyslipidemia (group II, *p* = 0.02; Table 14).

In patients with atrial fibrillation, statistically significant differences were observed for factor X concentrations on day 1 in the conservatively treated group, whereas no significant differences were found for protein Z or ZPI concentrations (group II, Table 15).

The results of PZ, ZPI, and FX in relation to patients’ neurological status, assessed using the NIHSS, are presented in Table 16. The type of treatment had a significant effect on NIHSS scores on day 7 compared with day 1.

In the first assessment, patients in group I had slightly higher NIHSS scores compared with group II (7.5 points vs. 5.5 points, respectively), but this difference was not statistically significant (*p* = 0.21). On day 7, patients in study group I had significantly lower NIHSS scores (Me = 2.0) compared with patients in group II (Me = 4.0), and this difference was statistically significant (*p* < 0.01). In group I, the average score reduction was 5.0 points, whereas in group II the reduction was smaller by 1.5 points, and this difference was statistically significant (*p* < 0.01).

It is worth noting that in group II, patients with an ASTRAL score ≥ 31 had significantly higher ZPI values on both day 1 and 7 compared with patients with an ASTRAL score < 31 (*p* = 0.05).

Correlation analysis revealed a slight negative relationship between PZ levels on day 7 and mRS scores, observed only in study group II (Table 17).

## 4. Discussion

The concentration of protein Z on the first day after the onset of stroke symptoms was significantly higher in study group I, treated with intravenous thrombolysis t-PA (median = 2810.05 ng/mL), compared with group II (median = 2178.50 ng/mL; *p* = 0.024). The higher protein Z concentration at stroke onset in patients qualified for t-PA therapy can be explained by the fact that in this group, the thrombus is in the process of formation, in which factor X and its active form Xa play an important role. This process is accompanied by the simultaneous inactivation of activated factor Xa, mediated by the protein Z–ZPI complex [9,10,14]. Thus, activation of the protein Z system occurs, manifested by higher serum levels. In contrast, in group 2, where more than 4.5 h had elapsed since stroke onset, the protein Z complex had already been consumed earlier, resulting in lower levels. It should be emphasized that the comparison between thrombolysis-treated patients and those managed conservatively reflects not only differences in treatment strategy but also differences in the time elapsed from stroke onset at the time of blood sampling, which may influence the observed biomarker profiles.

Similarly, on the seventh day after the onset of stroke symptoms, higher protein Z concentrations were observed in study group I (median = 2982.90 ng/mL) compared with study group II (median = 2286.50 ng/mL; *p* = 0.026). A higher level of the protein Z complex may suggest that the inactivation of factor Xa in patients treated with intravenous thrombolysis t-PA is more pronounced than in the non-thrombolysis group, which may have a beneficial effect on recovery in ischemic stroke patients.

No statistically significant differences in protein Z concentrations were observed between day 1 and day 7, either in study group 1 or in study group 2. This may indicate that, within such a short observation period, no meaningful changes in concentrations occur. It is possible that after 14 days, protein Z concentrations would differ, which would require further studies designed for a longer follow-up period.

Although a decrease in factor X (FX) concentration was observed on day 7 in the thrombolysis-treated group, this finding does not necessarily contradict the observed behavior of protein Z (PZ) and protein Z-dependent protease inhibitor (ZPI). The PZ–ZPI system regulates the activity of activated factor Xa rather than the total plasma concentration of factor X. Therefore, changes in FX concentration may reflect broader systemic or treatment-related effects during hospitalization, whereas alterations in PZ and ZPI levels are more closely related to the regulation of coagulation activity. The lack of parallel changes between FX concentrations and PZ/ZPI levels suggests that these parameters reflect different aspects of hemostatic regulation in the acute and subacute phases of ischemic stroke.

In the present study, no statistically significant associations were observed between protein Z or ZPI concentrations and common vascular comorbidities such as arterial hypertension, diabetes mellitus, or smoking status; however, the potential influence of these conditions on circulating protein Z and ZPI levels cannot be excluded and should be further explored in larger cohorts.

McQuillan et al. [12] also reported significantly higher protein Z levels in 173 patients with acute ischemic stroke (measured within seven days of stroke onset) compared with 186 individuals without stroke. The authors suggested that the elevated protein Z levels were related to endothelial cell activation and rupture of atherosclerotic plaques, processes characteristic of acute stroke.

Other studies [12,13,17,18] likewise demonstrated increased protein Z levels in the acute phase of ischemic stroke compared with patients examined in the convalescent phase, with protein Z concentrations in convalescent patients being similar to those observed in control subjects without stroke [12,17,18,19,20,21]. In line with these prior reports indicating elevated protein Z levels during the acute phase of ischemic stroke, we observed increased protein Z concentrations in our acute ischemic stroke cohort, with higher values in patients eligible for thrombolytic therapy compared with those managed conservatively. These findings suggest that protein Z elevation is a characteristic feature of the acute ischemic phase and may additionally reflect differences related to timing from stroke onset and treatment strategy within the acute stroke population.

It is noteworthy that in our cohort, patients treated with intravenous thrombolysis within 4.5 h (group I) had higher protein Z levels compared with patients managed conservatively, who were admitted more than 4.5 h after stroke onset (group II). This difference was statistically significant. The disparity in protein Z concentrations between groups I and II may reflect the release of this protein into the bloodstream during the early stages of ischemic stroke in response to endothelial injury or atherosclerotic plaque rupture, with the greatest increase likely occurring within the first hours of stroke. This requires further investigation.

By contrast, Słomka et al., in a meta-analysis of six clinical studies including a total of 1011 ischemic stroke patients and 1128 controls, did not identify such clear differences [13].

An interesting finding is the persistence of elevated protein Z concentrations on day 7 in patients of study group I, treated with t-PA, compared with study group II, managed conservatively. This phenomenon may be explained by the exposure of the damaged vascular intima (including endothelial cells) and/or ruptured atherosclerotic plaques during fibrinolysis, leading to the release of protein Z as a consequence of endothelial cell activation.

Tissue plasminogen activator (t-PA) induces the conversion of plasminogen to plasmin, which subsequently degrades cross-linked fibrin and fibrinogen into degradation products such as D-dimers, resulting in clot dissolution and vessel recanalization. Importantly, this pharmacological process does not involve mechanical injury to the vascular wall.

The elevated protein Z concentrations observed in patients treated with intravenous thrombolysis may therefore be related not to vascular wall damage, but rather to changes in the local hemostatic environment following clot dissolution, restoration of blood flow, and ongoing endothelial remodeling during the acute phase of ischemic stroke. Similar increases in circulating protein Z have been reported in clinical settings associated with altered endothelial–hemostatic balance, including postoperative states such as coronary artery bypass grafting; however, the underlying mechanisms in surgical and thrombolytic contexts are fundamentally different [22].

To our knowledge, no studies have been published comparing the behavior of protein Z concentrations in ischemic stroke patients treated with thrombolysis.

It is also noteworthy that protein Z-dependent protease inhibitor (ZPI) concentrations on the first day of stroke were significantly lower in patients treated with thrombolysis compared with those managed conservatively (median 5817.50 ng/mL vs. 11.452 ng/mL; *p* = 0.0021). A similar pattern was observed on day 7 (median 5545 ng/mL vs. 13.105 ng/mL; *p* = 0.0002).

The combination of significantly higher protein Z levels with simultaneously lower ZPI levels (*p* < 0.001) on day 1 may reflect the involvement of the protein Z–ZPI system in the regulation of activated factor Xa during the acute phase of ischemic stroke. As previously described by Han et al., protein Z, together with ZPI, inhibits activated factor Xa, which may lead to a relative consumption of ZPI under conditions of intensified coagulation–fibrinolysis interplay [9].

The elevated protein Z concentrations observed in patients in the acute phase of ischemic stroke, as well as on day 7 in those treated with intravenous thrombolysis (study group 1), should not be interpreted as an effect of thrombus formation induced by thrombolytic therapy. Rather, these findings may be associated with differences in the stage of the acute ischemic process, earlier admission after symptom onset, and exposure of the vascular wall following fibrinolysis, which may influence circulating protein Z levels—similar to observations reported after surgical vascular interventions [9].

## 5. Conclusions

In patients with acute ischemic stroke (AIS), significantly higher protein Z (PZ) concentrations were observed in the group treated with thrombolysis (patients with stroke symptoms < 4.5 h) compared with the conservatively treated group (patients with stroke symptoms > 4.5 h). This relationship could potentially be used in the assessment of patients presenting with stroke symptoms, for example, after nocturnal onset.

In thrombolysis-treated patients with dyslipidemia, higher protein Z values were noted on day 7 compared with day 1, whereas in patients without dyslipidemia, protein Z concentrations on day 7 were lower compared with day 1.

No correlations were observed between concentrations of protein Z, ZPI, or factor X and prognosis assessed using the NIHSS and ASTRAL scales. However, a slight negative correlation (r = −0.360, *p* = 0.043) was found between protein Z concentration and mRS score on day 7 in conservatively treated patients.

The assessment of protein Z in IS requires further clinical studies and may prove useful in the future for evaluating stroke severity and prognosis. Moreover, it may contribute to a more comprehensive understanding of the processes of coagulation, fibrinolysis, and fibrinolytic therapy in the acute phase of ischemic stroke. As new biomarkers of ischemic stroke are continuously being sought to improve understanding of its pathogenesis and to enhance diagnostic and prognostic assessment, protein Z and its associated complex may represent promising candidates in this regard.

## Figures and Tables

**Table 1:** Baseline characteristics of patients with acute ischemic stroke according to treatment strategy.

Variable	Thrombolysis Group	Conservative Group	*p*
Number of patients, *n*	52	32	–
Age, years, median (range)	70 (39–88)	70 (39–88)	0.72
Body weight, kg, median (range)	80 (55–130)	80 (56–136)	0.58
Stroke etiology (TOAST), *n* (%)			
Cardioembolic	12 (23.1)	12 (37.5)	0.34
Large-artery atherosclerosis	10 (29.2)	5 (15.6)	
Small-vessel disease	7 (13.5)	18 (18.8)	
Undetermined etiology	22 (44.2)	8 (28.1)	

**Table 2:** Comparison of protein Z (PZ) levels obtained on the first day in groups I and II (Me—median, Q1—first quartile, Q3—third quartile).

Variable	Study Group I (*n* = 52)			Study Group II (*n* = 32)			*p*
	Median	Q1	Q3	Median	Q1	Q3	
Protein Z [ng/mL]	2810	2037	3610	2178	1824	2832	0.024

**Table 3:** Comparison of protein Z-dependent protease inhibitor (ZPI) concentrations on the first day between groups I and II (Me—median, Q1—first quartile, Q3—third quartile).

Variable	Study Group I (*n* = 52)			Study Group II (*n* = 32)			*p*
	Median	Q1	Q3	Median	Q1	Q3	
ZPI [ng/mL]	5817	2554	11,916	11,452	6040	13,537	0.0021

**Table 4:** Comparison of factor X levels on the first day between study group I and control group II (Me—median, Q1—first quartile, Q3—third quartile).

Variable	Study Group I (*n* = 52)			Study Group II (*n* = 32)			*p*
	Median	Q1	Q3	Median	Q1	Q3	
FX [%]	118	103	132	117	108	110	0.478

**Table 5:** Comparison of protein Z concentrations on the seventh day between study group I and control group II (Me—median, Q1—first quartile, Q3—third quartile).

Variable	Study Group I (*n* = 52)			Study Group II (*n* = 32)			*p*
	Median	Q1	Q3	Median	Q1	Q3	
PZ [ng/mL]	2982	1806	3592	2286	1594	3074	0.026

**Table 6:** Comparison of protein Z-dependent protease inhibitor (ZPI) concentrations on the seventh day between study groups I and II (Me—median, Q1—first quartile, Q3—third quartile).

Variable	Study Group I (*n* = 52)			Study Group II (*n* = 32)			*p*
	Median	Q1	Q3	Median	Q1	Q3	
ZPI [ng/mL]	5545	2501	9521	13,105	6707	15,732	0.0002

**Table 7:** Comparison of factor X concentrations on the seventh day between study groups I and II (Me—median, Q1—first quartile, Q3—third quartile).

Variable	Study Group I (*n* = 52)			Study Group II (*n* = 32)			*p*
	Median	Q1	Q3	Median	Q1	Q3	
FX [%]	113	100	130	123	110	127	0.135

**Table 8:** Comparison of PZ, ZPI, and FX results between study groups I and II (Me—median, Q1—first quartile, Q3—third quartile).

Variables	Group I			Group II			*p*
	Me	Q1	Q3	Me	Q1	Q3	
PZ [Day 1—ng/mL]	2810	2037	3611	2179	1824	2832	0.024
PZ [Day 7—ng/mL]	2983	1807	3593	2286	1595	3074	0.026
PZ [Difference—ng/mL]	68.80	−205	264	40	−182	430	0.661
*p* for difference (Day 1 vs. Day 7)	*p* = 0.465			*p* = 0.329			
ZPI [Day 1—ng/mL]	5818	2555	11,916	11,453	6040	13,538	0.0021
ZPI [Day 7—ng/mL]	5545	2501	9522	13,105	6708	15,733	0.0002
ZPI [Difference—ng/mL]	−39	−1088	694	430	−776	1533	0.17
*p* for difference (Day 1 vs. Day 7)	*p* = 0.203			*p* = 0.531			
FX [Day 1—%]	118	103	132	118	108	110	0.478
FX [Day 7—%]	114	100	130	123	110	127	0.135
FX [Difference—%]	−3	−11	8	4	0.65	11	0.011
*p* for difference (Day 1 vs. Day 7)	*p* < 0.001			*p* < 0.001			

**Table 9:** Correlations between PZ, ZPI, and FX at the first and second measurements (day 1 and 7).

		PZ [ng/mL]	ZPI [ng/mL]	FX [%]
Variable (Day 7)	PZ [ng/mL]	-	r = −0.27 (*p* = 0.57)	r = 0.27 (*p* = 0.016)
	ZPI [ng/mL]	-	-	r = −0.15 (*p* = 0.185)
	FX [%]	-	-	-
Variable (Day 1)	PZ [ng/mL]	-	r = −0.38 (*p* < 0.001)	r = 0.26 (*p* = 0.021)
	ZPI [ng/mL]	-	-	r = −0.29 (*p* = 0.01)
	FX [%]	-	-	-

**Table 10:** Comparison of PZ results according to stroke etiology defined by the TOAST classification.

Group	Variables	*n*	PZ [Day 1—ng/mL]			PZ [Day 7—ng/mL]			PZ [Difference—ng/mL]		
			Me	Q1	Q3	Me	Q1	Q3	Me	Q1	Q3
Overall	cardioembolic	24	2545	1554	3295	2287	1497	3297	96	−298	289
	extracranial large-artery atherosclerosis	15	2222	1071	3169	1777	909	2809	−162	−523	133
	undetermined etiology	32	2863	1882	3403	3013	1884	3545	69	−167	339
	small-vessel disease	13	2714	2136	3587	3037	2361	3750	287	−46	389
	*p*		0.24			0.02			0.21		
Study I	cardioembolic	12	2545	1662	3346	2517	1497	3418	96	−340	254
	extracranial large-artery atherosclerosis	10	2412	1008	3169	2110	1583	2809	−145	−360	133
	undetermined etiology	23	2980	2020	3635	3050	2336	3595	69	−169	254
	small-vessel disease	7	3587	2739	3703	3750	3170	3950	201	−49	770
	*p*		0.16			0.01			0.37		
Study II	cardioembolic	12	2438	1554	3204	2287	1825	3275	95	−259	529
	extracranial large-artery atherosclerosis	5	1884	1829	2222	1632	909	1777	−162	−1023	−52
	undetermined etiology	9	2372	1820	2855	2728	1620	3206	24	−165	469
	small-vessel disease	6	2083	1989	2482	2339	1985	2523	380	−46	389
	*p*		0.94			0.36			0.41		

**Table 11:** Comparison of ZPI results according to stroke etiology defined by the TOAST.

Group	Variables	*n*	ZPI [Day 1—ng/mL]			ZPI [Day 7—ng/mL]			ZPI [Difference—ng/mL]		
			Me	Q1	Q3	Me	Q1	Q3	Me	Q1	Q3
Overall	cardioembolic	24	8190	5113	12,762	8468	4174	15,635	223	−665	3075
	extracranial large-artery atherosclerosis	13	12,075	5205	16,135	11,466	5090	15,115	−607	−2985	0
	undetermined etiology	31	6695	3208	13,450	8011	2816	13,925	430	−1241	1567
	small-vessel disease	13	5990	2811	6520	4838	2917	6095	−435	−967	390
	*p*		0.11			0.16			0.38		
Study I	cardioembolic	12	5634	2724	7190	4968	2703	7233	96	−790	1347
	extracranial large-artery atherosclerosis	10	10,801	2635	16,015	6465	2333	13,000	−705	−3864	−606
	undetermined etiology	23	6535	2555	12,947	7096	2565	12,211	295	−1395	1569
	small-vessel disease	7	2811	2112	6265	2917	2376	5545	−435	−720	550
	*p*		0.07			0.33			0.39		
Study II	cardioembolic	12	11,513	9115	13,490	14,233	10,673	16,163	458	−440	5085
	extracranial large-artery atherosclerosis	5	12,765	12,480	16,135	15,115	13,150	17,070	775	−115	2635
	undetermined etiology	9	11,290	5375	13,450	10,655	6985	15,040	575	385	1455
	small-vessel disease	6	6305	5990	10,370	6635	4838	9980	−679	−1190	−40
	*p*		0.45			0.14			0.56		

**Table 12:** Comparison of FX results according to stroke etiology defined by the TOAST classification.

Group	Variables	*n*	FX [Day 1—%]			FX [Day 7—%]			FX [Difference—%]		
			Me	Q1	Q3	Me	Q1	Q3	Me	Q1	Q3
Overall	cardioembolic	23	120.60	106.60	128.70	122.30	107.20	132.50	2.65	−6.40	8.45
	extracranial large-artery atherosclerosis	15	111.43	96.21	135.50	107.70	99.08	124.20	−3.69	−14.76	8.44
	undetermined etiology	30	118.00	103.70	129.80	118.10	101.61	126.80	0.00	−7.35	7.20
	small-vessel disease	13	112.20	103.70	126.20	123.50	106.40	131.90	6.60	−5.00	11.90
	*p*		0.9			0.41			0.24		
Study I	cardioembolic	12	106.60	95.17	130.80	107.20	91.53	129.10	−1.15	−8.33	6.90
	extracranial large-artery atherosclerosis	10	112.76	96.21	138.30	109.17	81.44	121.90	−12.68	−15.00	1.64
	undetermined etiology	23	124.80	98.22	134.47	117.40	100.61	130.40	0.00	−7.90	8.10
	small-vessel disease	7	120.80	109.50	131.00	131.90	106.40	135.50	−3.10	−11.90	22.00
	*p*		0.86			0.35			0.45		
Study II	cardioembolic	12	122.00	113.55	128.65	127.30	119.25	133.15	4.80	1.95	8.45
	extracranial large-artery atherosclerosis	5	105.50	99.71	119.70	107.70	106.70	124.20	3.59	2.20	15.05
	undetermined etiology	9	117.30	103.70	124.00	119.10	113.00	126.80	0.00	−1.50	1.10
	small-vessel disease	6	107.95	103.60	120.50	118.10	105.40	124.10	8.35	1.80	11.90
	*p*		0.11			0.37			0.51		

**Table 13:** Comparison of PZ results according to the presence of dyslipidemia.

Group	Variables	*n*	PZ [Day 1—ng/mL]			PZ [Day 7—ng/mL]			PZ [Difference—ng/mL]		
			Me	Q1	Q3	Me	Q1	Q3	Me	Q1	Q3
Overall	No dyslipidemia	14	2123	1820	3026	2252	1613	3050	−181	−734	389
	Dyslipidemia	70	2670	1989	3484	2924	1766	3534	69	−165	323
	*p*		0.24			0.09			0.32		
Study I	No dyslipidemia	6	2592	2302	3286	2409	1613	3050	−347	−909	133
	Dyslipidemia	46	2895	2020	3635	3067	1847	3682	78	−169	275
	*p*		0.69			0.16			0.03		
Study II	No dyslipidemia	8	1906	1683	2485	2252	1264	2850	113	−467	568
	Dyslipidemia	24	2307	1909	2832	2290	1601	3074	40	−145	389
	*p*		0.38			0.57			0.82		

**Table 14:** Comparison of ZPI results according to the presence of dyslipidemia.

Group	Variables	*n*	ZPI [Day 1—ng/mL]			ZPI [Day 7—ng/mL]			ZPI [Difference—ng/mL]		
			Me	Q1	Q3	Me	Q1	Q3	Me	Q1	Q3
Overall	No dyslipidemia	13	10,625	4831	12,480	11,760	5818	15,575	475	−40	5255
	Dyslipidemia	68	6618	3539	13,074	7114	3317	13,333	−74	−1190	775
	*p*		0.93			0.38			0.09		
Study I	No dyslipidemia	5	2555	2492	10,625	2786	2172	5818	−383	−705	295
	Dyslipidemia	44	6041	2635	11,995	5643	2533	9661	−19	−1088	694
	*p*		0.59			0.65			1		
Study II	No dyslipidemia	8	11,375	7625	12,623	14,520	8855	16,645	1872	388	6370
	Dyslipidemia	24	11,505	5948	15,690	11,858	6708	15,733	−113	−1217	788
	*p*		0.65			0.34			0.02		

**Table 15:** Comparison of FX results between patients with and without atrial fibrillation (Me—median, Q1—first quartile, Q3—third quartile).

Group	Variables	*n*	FX [Day 1—% ]			FX [Day 7—% ]			FX [Difference—%]		
			Me	Q1	Q3	Me	Q1	Q3	Me	Q1	Q3
Overall	No atrial fibrillation	61	117.30	103.60	129.70	117.50	103.30	126.80	0.20	−7.90	10.10
	Atrial fibrillation	20	120.90	108.55	128.65	124.25	107.20	130.80	2.50	−5.50	7.30
	*p*		0.58			0.5			0.92		
Study I	No atrial fibrillation	43	120.60	100.90	134.47	117.40	100.61	131.90	−3.10	−11.90	9.30
	Atrial fibrillation	8	106.50	100.18	129.60	108.20	93.78	121.50	−2.30	−7.30	0.60
	*p*		0.62			0.53			0.34		
Study II	No atrial fibrillation	18	113.35	103.60	120.50	118.30	106.70	125.90	2.00	−0.75	13.48
	Atrial fibrillation	12	122.00	113.55	128.65	127.30	119.25	133.15	4.80	1.95	8.45
	*p*		0.03			0.08			0.52		

**Table 16:** Correlations of NIHSS scores on day 1 and day 7 with PZ, ZPI, and FX results.

Variable Pair		Group 1 and 2		Group 1		Group 2	
		r	*p*	r	*p*	r	*p*
NIHSS I	PZ [Day 1—ng/mL]	0.052	0.653	0.166	0.259	−0.209	0.268
	PZ [Day 7—ng/mL]	−0.039	0.734	0.114	0.439	−0.317	0.088
	ZPI [Day 1—ng/mL]	−0.050	0.664	−0.184	0.211	0.260	0.165
	ZPI [Day 7—ng/mL]	−0.065	0.572	−0.220	0.133	0.291	0.118
	FX [Day 1—%]	0.015	0.898	0.054	0.718	−0.064	0.735
	FX [Day 7—%]	−0.001	0.994	0.118	0.425	−0.215	0.254
NIHSS II	PZ [Day 1—ng/mL]	−0.056	0.626	0.092	0.533	−0.267	0.155
	PZ [Day 7—ng/mL]	−0.068	0.555	0.143	0.332	−0.362	0.049
	ZPI [Day 1—ng/mL]	0.035	0.761	−0.088	0.552	0.150	0.429
	ZPI [Day 7—ng/mL]	0.053	0.647	−0.104	0.483	0.240	0.202
	FX [Day 1—%]	−0.032	0.783	−0.002	0.989	−0.118	0.536
	FX [Day 7—%]	0.012	0.916	0.099	0.503	−0.361	0.050

**Table 17:** Correlations between PZ values and mRS at the first and second measurements.

Variable Pair		Group 1 and 2		Group 1		Group 2	
		r	*p*	r	*p*	r	*p*
PZ [Day 1—ng/mL]	mRS (first measurement)	−0.129	0.241	−0.140	0.323	−0.247	0.172
	mRS (second measurement)	−0.100	0.364	−0.027	0.848	−0.176	0.334
	mRS (difference II–I)	0.016	0.882	0.106	0.454	0.078	0.671
PZ [Day 7—ng/mL]	mRS (first measurement)	−0.115	0.296	0.296	0.775	−0.335	0.061
	mRS (second measurement)	−0.179	0.103	0.103	0.778	−0.360	0.043
	mRS (difference II–I)	−0.092	0.403	0.403	0.956	−0.055	0.767
PZ [Difference—ng/mL]	mRS (first measurement)	0.017	0.875	0.214	0.127	0.127	0.357
	mRS (second measurement)	−0.143	0.194	−0.025	0.859	0.859	0.120
	mRS (difference II–I)	−0.189	0.085	−0.243	0.082	0.082	0.386

## Data Availability

Data is contained within the article.
